# Biophysical characterization of hit compounds for mechanism-based enzyme activation

**DOI:** 10.1371/journal.pone.0194175

**Published:** 2018-03-16

**Authors:** Xiangying Guan, Alok Upadhyay, Sudipto Munshi, Raj Chakrabarti

**Affiliations:** Division of Fundamental Research, Chakrabarti Advanced Technology, Mount Laurel, New Jersey, United States of America; Università degli Studi di Milano, ITALY

## Abstract

Across all families of enzymes, only a dozen or so distinct classes of non-natural small molecule activators have been characterized, with only four known modes of activation among them. All of these modes of activation rely on naturally evolved binding sites that trigger global conformational changes. Among the enzymes that are of greatest interest for small molecule activation are the seven sirtuin enzymes, nicotinamide adenine dinucleotide (NAD^+^)-dependent protein deacylases that play a central role in the regulation of healthspan and lifespan in organisms ranging from yeast to mammals. However, there is currently no understanding of how to design sirtuin-activating compounds beyond allosteric activators of SIRT1-catalyzed reactions that are limited to particular substrates. Here, we introduce a general mode of sirtuin activation that is distinct from the known modes of enzyme activation. Based on the conserved mechanism of sirtuin-catalyzed deacylation reactions, we establish biophysical properties of small molecule modulators that can in principle result in enzyme activation for diverse sirtuins and substrates. Building upon this framework, we propose strategies for the identification, characterization and evolution of hits for mechanism-based enzyme activating compounds.

## Introduction

The silent information regulator proteins (sirtuins) have emerged as critical regulators of many cellular pathways. In particular, these enzymes protect against age-related diseases and serve as key mediators of longevity in evolutionarily distant organismic models [1]. Sirtuins are NAD^+^-dependent lysine deacylases, requiring the cofactor NAD^+^ to cleave acyl groups from lysine side chains of their substrate proteins, and producing nicotinamide (NAM) as a by-product. A thorough understanding of sirtuin chemistry is not only of fundamental importance, but also of considerable medicinal importance, since there is enormous interest in the development of new mechanism-based sirtuin modulators [2, 3]. The mechanism of sirtuin-catalyzed deacylation is depicted in S1 Fig [4–9].

Recently, in order to extend mammalian healthspan and lifespan, intense interest has developed in the activation of the seven mammalian sirtuin enzymes (SIRT1-7). Prior work on sirtuin activation has relied exclusively on experimental screening, with an emphasis on allosteric activation of the SIRT1 enzyme. Indeed, small molecule allosteric activators of SIRT1 have been demonstrated to induce lifespan extension in model organisms such as mice [10, 11]. Allosteric activation is one of four known modes by which small molecules can activate enzymes [12]. Allosteric activators most commonly function by decreasing the dissociation constant for the substrate (the acylated protein dissociation constant *K*_*d*,*Ac*−Pr_ in the case of sirtuins).

Nearly all known sirtuin activators allosterically target SIRT1, work with a limited set of substrates [13–18], and bind outside of the active site to an allosteric domain in SIRT1 that is not shared by SIRT2-7 [19]. It is now known that other sirtuins—including SIRT2, SIRT3 and SIRT6—and multiple protein substrates play significant roles in regulating mammalian longevity [20–22]. General strategies for the activation of any mammalian sirtuin (including activation of SIRT1 for other substrates) are hence of central importance, but not understood.

Foundations for the rational design of mechanism-based sirtuin activators have been lacking, partly due to the absence of a clear understanding of the kinetics of sirtuin-catalyzed deacylation. Several types of mechanism-based sirtuin inhibitors have been reported recently in the literature, including Ex-527 and Sir-Real2 [23–25]. However, mechanism-based activation has proven far more elusive, due to the difficulty in screening for the balance of properties needed for a modulator to have the net effect of accelerating catalytic turnover.

In this regard, unlike allosteric activators like resveratrol, which are SIRT1-specific and have not been successfully applied to other sirtuins [19], NAD^+^ supplementation [26] can activate most mammalian sirtuins in a substrate-independent fashion. The effects of NAD^+^ supplementation are not specific to sirtuins and prohibitively high concentrations of NAD^+^, along with associated undesirable side effects, may be required to elicit the increases in sirtuin activity required to combat age-related diseases. A preferred general strategy for activation of sirtuins (S1 Fig) would be to lower the *K*_*m*_ for NAD^+^ (*K*_*m*,*NAD*+_). *K*_*m*,*NAD*+_ reduction would have a similar activating effect to NAD^+^ supplementation, but would be selective for sirtuins and could potentially even provide isoform specific sirtuin activation. Importantly, due to the sirtuin nicotinamide cleavage reaction that involves the NAD^+^ cofactor, modulation of *K*_*m*,*NAD*+_ may in principle be achievable by means other than altering the binding affinity of NAD^+^. Unlike allosteric activation that reduces *K*_*d*,*Ac*−Pr_, this approach could be applicable to multiple sirtuins and substrates.

Several compounds have been reported in the literature as being activators of sirtuins other than SIRT1 [27–30]. However, some of the aforementioned compounds have not been characterized using initial rate assays [27, 29] and others have been studied using only labeled activity assays [29] that may be susceptible to false positives. Very little is known about the mechanistic functioning of these activators. However, such an understanding is critical to the rational design of mechanism-based activators.

In this paper, we present a general framework for activation of sirtuin enzymes that is distinct from any of the known modes of enzyme activation. We first introduce a steady-state model of sirtuin-catalyzed deacylation reactions in the presence of NAD^+^ cofactor and endogenous inhibitor NAM, and then establish quantitatively how k_cat_*/K*_*m*,*NAD+*_ can be modified by small molecules, identifying the biophysical properties that small molecules must have to function as such mechanism-based activators. The principles introduced can also be generalized to the reduction of peptide substrate *K*_*m*_ through non-allosteric mechanisms. We propose strategies suitable for designing mechanism-based sirtuin activating compounds (MB-STACs), and describe how to characterize sirtuin modulators in order to determine whether they possess the proposed characteristics of MB-STACs presented.

## Results

### Steady-state sirtuin kinetic modeling

To a greater extent than inhibitor design, rational activator design requires the use of a mechanistic model in the workflow. In this section we develop a steady state model for sirtuin-catalyzed deacylation that is suitable for a) investigation of the mode of action of mechanism-based sirtuin modulators, including activators; b) design of MB-STACs. We first summarize the state of knowledge regarding the sirtuin-catalyzed deacylation mechanism.

The sirtuin catalytic cycle (S1 Fig) is believed to proceed in two consecutive stages [4]. The initial stage (ADP-ribosylation) involves the cleavage of the NAM moiety of NAD^+^ and the nucleophilic attack of the acyl-Lys side chain of the protein substrate to form a positively charged O-alkylimidate intermediate [4, 9]. NAM-induced reversal of the intermediate (the so-called base exchange reaction) causes reformation of NAD^+^ and acyl-Lys protein. The energetics of this reversible reaction affects both the potency of NAM inhibition of sirtuins and the Michaelis constant for NAD^+^ (*K*_*m*,*NAD+*_). The second stage of sirtuin catalysis, which includes the rate-determining step, involves four successive steps that culminate in deacylation of the Lys side chain of the protein substrate and the formation of O-acetyl ADP ribose coproduct [4, 6, 31].

A tractable steady state model suitable for the purpose of mechanism-based sirtuin activator design must account for the following important features:

- The calculated free energy of activation for NAM cleavage (ADP -ribosylation of the acyl-Lys substrate) in the bacterial sirtuin enzyme Sir2Tm as computed through mixed quantum/molecular mechanics (QM/MM) methods is 15.7 kcal mol^-1^ [5, 32]. An experimental value of 16.4 kcal mol^-1^ for the activation barrier in the yeast sirtuin homolog Hst2 was estimated from the reaction rate 6.7 s^-1^ of NAM formation. The NAM cleavage reaction is endothermic, with a computed Δ*G* of 4.98 kcal mol^-1^ in Sir2Tm [32].
- The calculated free energy of activation for the rate limiting chemistry step (collapse of the bicyclic intermediate) from QM/MM simulations is 19.2 kcal mol^-1^ for Sir2Tm [33], in good agreement with the experimental value of 18.6 kcal mol^-1^ estimated from the k_cat_ value of 0.170 ± 0.006 s^-1^ [34] (0.2 ± 0.03 s^-1^ for Hst2 [9]). We note that the relative magnitudes of the rate constants for the two slowest chemistry steps may vary for other sirtuins, like mammalian sirtuins. For some sirtuins, product release may be rate limiting.
- The remaining steps in the catalytic cycle are significantly faster than the above steps. The other chemistry steps in stage 2 of the reaction are effectively irreversible [33], as is product release in the presence of saturating peptide concentrations.

We hence include in our kinetic model representations of all steps in stage 1 of the reaction, including the NAM cleavage/base exchange and NAM binding steps. However, for simplicity, we do not include in the present model a representation of each of the individual chemistry steps in stage 2 of the reaction or final product release, instead subsuming these steps under the smallest rate constant, which we call k_cat_. Since all these steps are effectively irreversible, the full steady state model including these steps can be immediately derived from the basic model through simple modifications, to be described in a subsequent revision, that are not essential to the analysis of mechanism-based activation. The above observations motivate the kinetic model represented in S2 Fig [35]. S2 Fig shows a general reaction scheme for sirtuin deacylation including base exchange inhibition.

Because of the physiological benefits of improving catalytic efficiency under NAD^+^ depletion rather than peptide depletion conditions, we assume saturating peptide conditions in our kinetic modeling in this paper. However, precisely analogous equations could be derived for saturating NAD^+^, in the event that activation under peptide depletion conditions is desired. We previously presented a preliminary theoretical and computational study of the mechanism by which NAM affects sirtuin activity [35]. In the present work, we build upon that foundation to establish a theoretical model for mechanism-based sirtuin modulators, including activators, in the presence of arbitrary concentrations of NAD^+^ and NAM. The reaction mechanism of sirtuins precludes the use of rapid equilibrium methods for the derivation of even an approximate initial rate model; steady-state modeling is essential. The rate equations for the reaction network in S2 Fig enable the derivation of steady-state conditions for the reaction. Solving the linear system of algebraic steady-state equations and mass balance constraints for the concentrations [*E*^.^*Ac-Pr*], [*E*^.^*Ac-Pr*^.^*NAD*^+^], [*E*^.^*ADPR-Ac-Im*^.^*NAM*], [*E*^.^*ADPR-Ac-Im*], [*E*^.^*NAM*] in terms of the rate constants and [NAD^+^], [NAM], which are assumed to be in significant excess and hence approximately equal to their initial concentrations [NAD^+^]_0_, [NAM]_0_ respectively, we obtain expressions of the form (1):

$$\begin{array}{l} \left[E.Ac-\text{Pr}]/{\left[E\right]}_{0}=c_{11}+c_{12}[\text{NAM}\right]\\ \left[E.Ac-\text{Pr}.\text{NA}{\text{D}}^{+}]/{\left[E\right]}_{{}_{0}}=c_{21}[NAD^{+}]+c_{22}[\text{NA}{\text{D}}^{+}][\text{NAM}\right]\\ \left[E.ADPR-Ac-\text{Im}.NAM]/{\left[E\right]}_{0}=c_{31}[NAD^{+}]+c_{32}[NAD^{+}][NAM\right]\\ \left[E.ADPR-Ac-\text{Im}]/{\left[E\right]}_{0}=c_{41}[\text{NA}{\text{D}}^{+}\right]\\ {}[E.\text{Ac}-\text{Pr}.NAM]/{\left[E\right]}_{0}=c_{51}[NAD^{+}]+c_{52}^{}[NAM]+c_{53}[\text{NA}{\text{D}}^{+}][\text{NAM}]+c_{54}{\left[NAM\right]}^{2} \end{array}$$ (1)

where the term c_54_ that is second order in [NAM] will be omitted from the analysis below. Expressions for the c_ij_’s are provided in the Appendix A2. The initial rate of deacylation can be then expressed

$$\frac{v}{v_{\text{max}}}=\frac{[NAD^{+}]\left(1+\frac{\left[NAM\right]}{K_{1}}\right)}{K_{m,NAD+}\left(1+\frac{\left[NAM\right]}{K_{2}}\right)+[NAD^{+}]\left(1+\frac{\left[NAM\right]}{K_{3}}\right)}$$ (2)

where the definitions of the steady state constants in terms of fundamental rate constants in the enzymatic reaction mechanism are presented in Appendix A1. *K*_*ex*_ = k_-2_/k_2_, and the approximations refer to the case where *k*_*4*_ *<< k*_*j*_, *j ≠ 4*. The quality of this approximation can be assessed for the chemistry steps based on QM/MM simulation data, which was cited above for yeast and bacterial sirtuins, or from experimental methods for the estimation of all rate constants in the model (see below). Expression (2) can be used to calculate the initial rate of sirtuin-catalyzed deacylation for specified intracellular concentrations of NAD^+^ and NAM, assuming the rate constants are known.

Eq (2) is typically represented graphically in terms of either double reciprocal plots at constant [NAM] or Dixon plots at constant [NAD^+^]. In the former case, the slope of the plot (1/v *vs* 1/ [NAD^+^]) at [NAM] = 0 is $K_{m,NAD^{+}}/v_{\text{max}}$, for which the expression is:

$$\frac{K_{m,NAD^{+}}}{v_{\text{max}}}=\frac{1}{{\left[E\right]}_{0}}\left(\frac{1}{k_{1}}+K_{d,NAD+}\frac{k_{-3}+k_{-2}}{k_{-3}k_{2}}\right)=\frac{K_{m,NAD^{+}}}{k_{cat}{\left[E\right]}_{0}}$$ (3)

whereas for the Dixon plot, the expression for the slope at 1/[NAD^+^] = 0 is approximately [35, 36]:

$$\frac{1}{K_{3}}\frac{1}{v_{max}}\approx \frac{1+K_{ex}}{K_{d,NAM}}\;\frac{1}{k_{cat}{\left[E\right]}_{0}}$$ (4)

We note that Eq (3) for catalytic efficiency applies irrespective of the small k_4_ approximation. The steady state parameter *α* Eq (15) in Appendix A1, which is a measure of the extent of competitive inhibition by the endogenous inhibitor NAM against the cofactor NAD^+^, can be expressed in terms of the ratio of *K*_*d*,*NAD+*_ and *K*_*m*,*NAD+*_ [35, 36]:

$$\alpha =\frac{K_{3}}{K_{2}}\approx \frac{K_{d,NAD+}}{K_{m,NAD+}}\frac{K_{ex}^{}}{1+K_{ex}}$$ (5)

which, together with expression (12) in Appendix A1 for *K*_*m*,*NAD+*_, demonstrates how the kinetics of inhibition of deacylation by NAM can reveal differences in NAD^+^ binding affinity and NAM cleavage rates among sirtuins. In addition to the approximation *k*_*4*_ *<< k*_*j*_, *j ≠ 4*, several experimental observations can further simplify the form of the expressions for the sirtuin steady state constants. First, we assume *k*_*-3*_ *>> k*_*j*_, *j ≠ -2* based on viscosity measurements that suggest NAM dissociates rapidly following cleavage [37]. Under this approximation, the expression for *K*_*m*,*NAD+*_ becomes:

$$K_{m,NAD+}\approx k_{4}\left(\frac{1}{k_{1}}+\frac{K_{d,NAD+}}{k_{2}}\right)$$ (6)

Such approximations will be studied in greater detail in a subsequent work.

**Fig 1:**
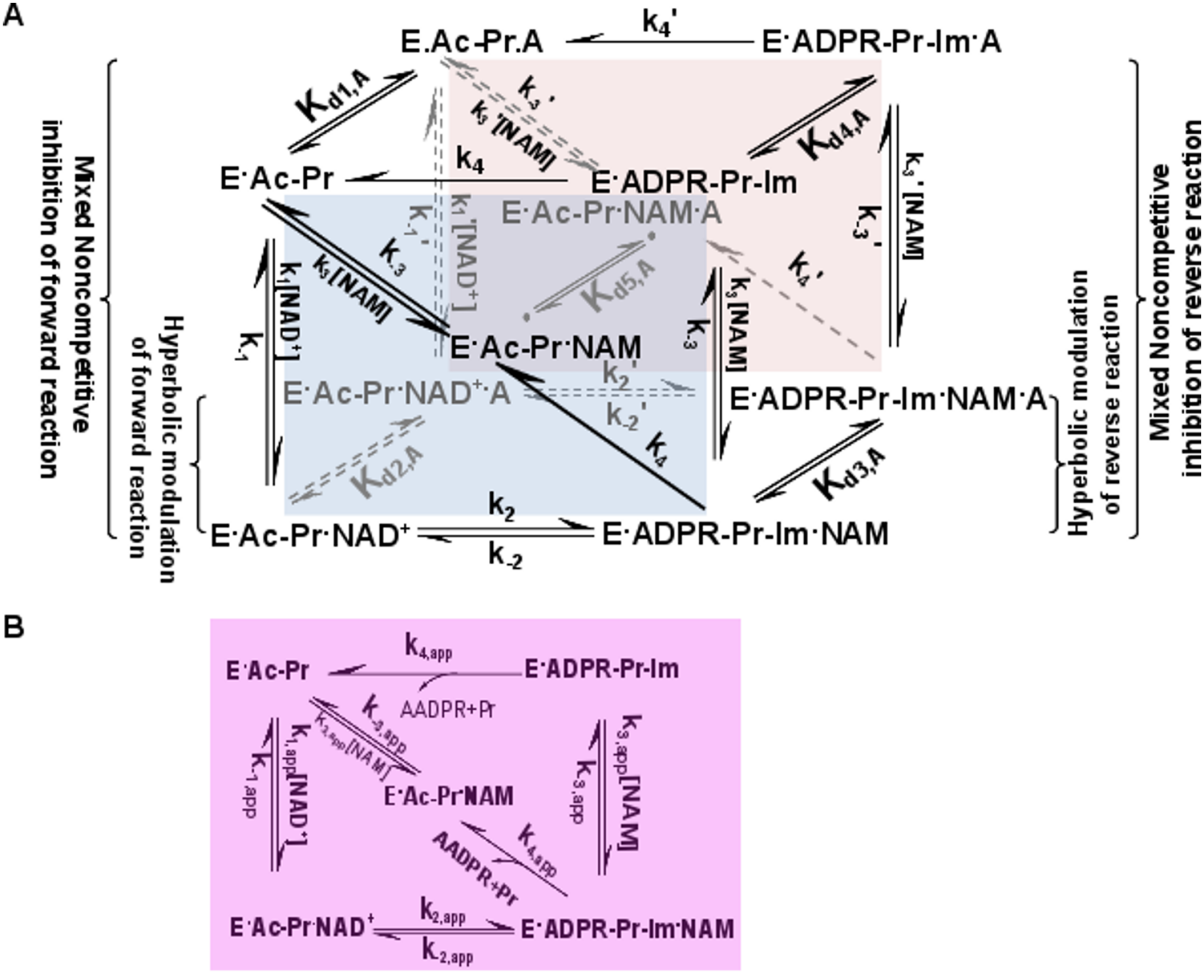
General model for mechanism-based sirtuin enzyme activation. **(A)** The front face of the cube (blue) depicts the salient steps of the sirtuin reaction network in the absence of bound modulator. The back face of the cube (red) depicts the reaction network in the presence of bound modulator (denoted by “A”). Each rate constant depicted on the front face has an associated modulated value on the back face, designated with a prime, which is a consequence of modulator binding. **(B)** The purple face is the apparent reaction network in the presence of a nonsaturating concentration of modulator.

As can be seen from Fig 1B, the kinetics of the NAM cleavage reaction and the rate limiting step of deacylation both play essential roles in determining the value of *K*_*m*,*NAD+*_. Note that in rapid equilibrium models of enzyme kinetics, which are not applicable to sirtuins, *K*_m_ ≈ *K*_*d*_. The difference between *K*_*d*,*NAD+*_ and *K*_*m*,*NAD+*_ has important implications for mechanism-based activation of sirtuins by small molecules [35]. In particular, as we will show in this work, decrease of *K*_*m*,*NAD+*_ independently of *K*_*d*,*NAD+*_ can increase the activity of sirtuins at [NAM] = 0. The kinetic model above establishes foundations for how this can be done.

### Mechanism-based sirtuin activation

A prerequisite for enzyme activation is that the modulator must co-bind with substrates—NAD^+^ and acylated peptide in the case of sirtuins. Within the context of enzyme inhibition, two modes of action display this property: noncompetitive and uncompetitive inhibition. In the case of sirtuins, examples of noncompetitive inhibitors include SirReal2 [24], whereas examples of uncompetitive inhibitors include Ex-527 [23]. Though some known sirtuin inhibitors may satisfy the requirement of cobinding with substrates, they do not possess other critical attributes necessary for mechanism-based enzyme activation. While such compounds may have promising properties as potential hits for the development of mechanism-based activators, prior studies have only characterized their kinetic effects in terms of traditional rapid equilibrium formulations of enzyme inhibition, rather than a steady-state formulation for mechanism-based enzyme modulation. Although some previous work have begun to explore the mechanisms by which small molecules may activate sirtuins other than SIRT1 [38], further work aimed at establishing a foundation for the characterization and design of such compounds is needed.

Previous attempts to develop a general approach to sirtuin activation [37, 39] only considered competitive inhibitors of base exchange, which cannot activate in the absence of NAM. This is not actually a form of enzyme activation, but rather derepression of inhibition. Such derepression modalities based on competitive inhibition of product binding cannot be hits for activator design, since these compounds or their relatives cannot cobind with substrates. By contrast, here we present paradigms and design criteria for activation of sirtuins in either the absence or presence of NAM. Based on expression (3b) for *K*_*m*,*NAD+*_, it is in principle possible to activate sirtuins (not just SIRT1) for any substrate by alteration of rate constants in the reaction mechanism other than k_1_, k_-1_ and k_cat_, so as to reduce *K*_*m*,*NAD+*,_ not only *K*_*d*,*Ac-Pr*_ as with allosteric activators, which increase the peptide binding affinity of SIRT1 in a substrate-dependent fashion. We now explore how this may be achieved by augmenting the kinetic model to include putative mechanism-based activators (A) that can bind simultaneously with NAD^+^ and NAM. Fig 1 depicts the reaction diagram for mechanism-based activation of sirtuins. Note that only the top and front faces of this cube are relevant to the mechanism of action of the previously proposed competitive inhibitors of base exchange [37, 39]. Due to the novelty of the theoretical results on mechanism-based activation and their central role in the analysis that follows, the derivations of these results and associated approximations are provided.

At any [A] there exist apparent values of each of the rate constants in the sirtuin reaction mechanism. These are denoted by “app” in the Fig 1. There are also corresponding “app” values of the steady state, Michaelis, and dissociation constants in Eq (3). Moreover, at saturating [A] of a known activator, the modulated equilibrium and dissociation constants (which do not depend on determination of steady state species concentrations) can be estimated with only deacylation experiments according to the theory presented above. The exchange equilibrium constant (*K*_*ex*_') and NAD^+^, NAM dissociation constants (*K*_*d*,*NAD*+_′ and *K*_*d*,*NAM*_′) in the presence of A are related to their original values as follows:

$$K_{d,NAD+}\prime =K_{d,NAD+}\frac{K_{d2,A}}{K_{d1,A}};\;K_{ex}\prime =K_{ex}\frac{K_{d3,A}}{K_{d2,A}};\;K_{d,NAM}\prime =K_{d,NAM}\frac{K_{d3,A}}{K_{d4,A}}$$ (7)

where the *K*_*d*,*A*_’s are the dissociation constants for A depicted in Fig 1.

In order to predict the effect on $K_{m,NAD^{+},app}$ of a modulator with specified relative binding affinities for the complexes in the sirtuin reaction mechanism, it is important to develop a model that is capable of quantifying, under suitable approximations, the effect of such a modulator on the apparent steady state parameters of the enzyme. Since the full steady state expression relating the original to the apparent rate constants has many terms containing products of additional side and back face rate constants, we use a rapid equilibrium segments approach to arrive at simple definitions of the apparent Michaelis constant and other steady state constants for the reaction in terms of the original expressions for these constants and the dissociation constants for binding of A to the various complexes in the sirtuin reaction mechanism. This provides a minimal model with the least number of additional parameters required to model sirtuin activation mechanisms. In our treatment, we will assume that rapid equilibrium applies on both the side faces and the back face. Under this approximation, at low [A] the expressions for the induced changes in each of the rate constant products appearing in the coefficients c_ij_ and c_i’j’_, i’ = i of (Eq 1) (see Appendix A2 for expressions for these products) are the same and linear in [A]. For example, in the case of *E*.*Ac* − Pr, the steady state species concentrations become:

$$\begin{array}{l} \left[E.Ac-\text{Pr}]/{\left[E.Ac-\text{Pr}\right]}_{0}\approx c_{11}+c_{12}[\text{NAM}\right]\\ {}[E.Ac-\text{Pr}.\text{A}]/{\left[E.Ac-\text{Pr}\right]}_{0}\approx \frac{\left[A\right]}{K_{d1,A}}\left(c_{11}+c_{12}[\text{NAM}]\right) \end{array}$$ (8)

The rapid equilibrium segments expressions for all species concentrations in Eq (1) in the presence of A are provided in the S1 File. Expressions for apparent values of all steady state parameters introduced in Appendix A1 (i.e., modulated versions of constants *v*_max_, *K*_*m*_,_*NAD*+_, *K*_1_, *K*_2_, *K*_3_) in the presence of a given [A] can now be derived. In the following, several types of approximations will be invoked:

1. rapid equilibrium segments approximation
2. $k_{4}(1+K_{dl,A})\ll k_{j}(1+K_{dl^{\prime },A}),\;j\neq 4,\;l=1,\;\ldots ,\;5$
3. $k_{-3}(1+K_{dl,A})\gg k_{j}(1+K_{dl^{\prime },A}),\;j\neq -3,\;l=1,\;\ldots ,\;5$
  (rapid NAM dissociation)

**Fig 2:**
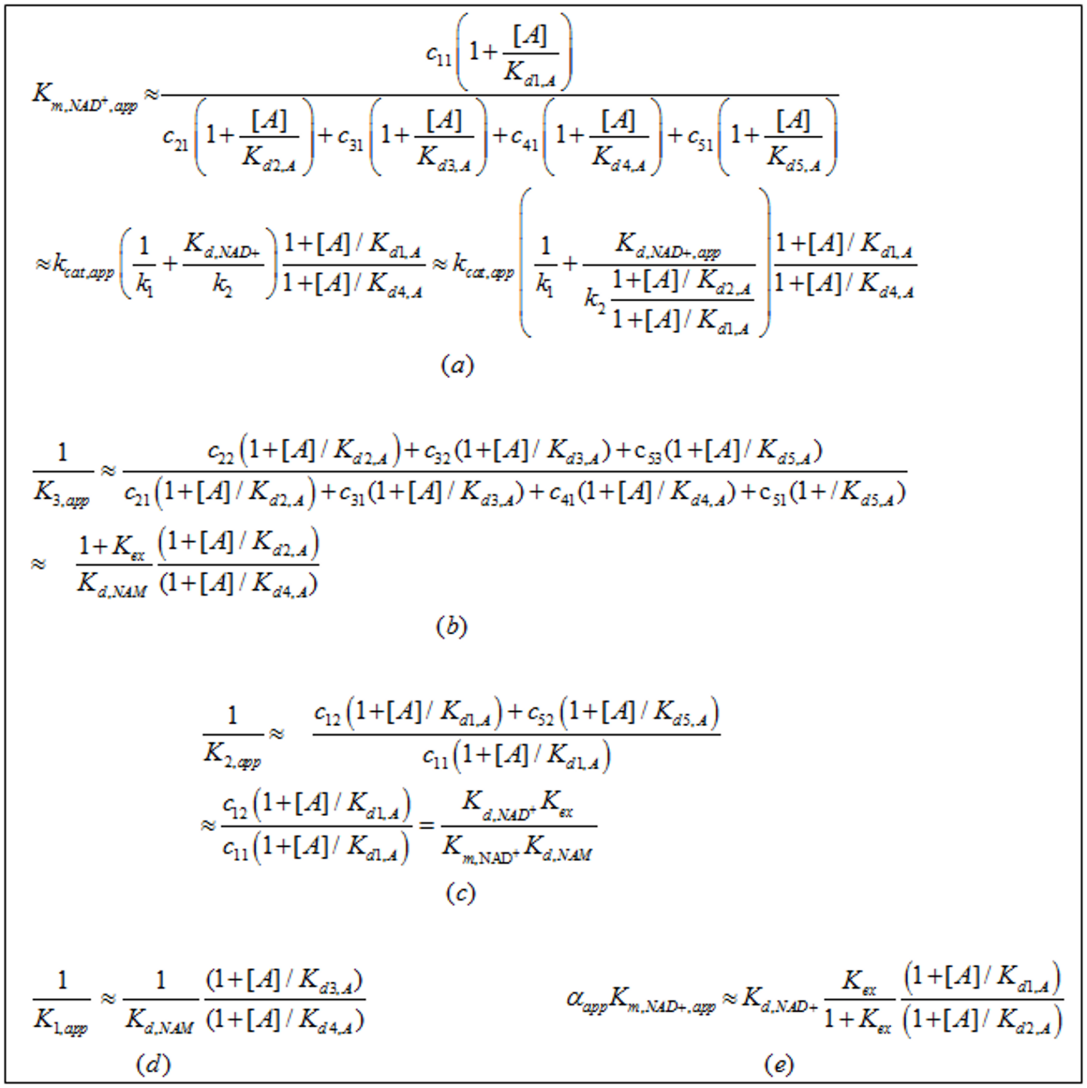
Modulated expressions for the steady state constants in Appendix A1 according to the rapid equilibrium segments approximation. c_ij_ expressions are provided in the Appendix A2. In particular, we emphasize that assumption (ii) may not hold for several mammalian sirtuins, but we apply this approximation to simplify the equations and provide physical insight. The general equations without this approximation can readily be derived using the principles introduced.

Fig 2 displays the expressions for each of the modulated steady state constants in Appendix A1 according to the rapid equilibrium segments approximation. Note that

$$\frac{v_{\text{max},app}}{{\left[E\right]}_{0}}=k_{cat,app}=\frac{\left(c_{31,app}+c_{41,app}\right)}{c_{21,app}+c_{31,app}+c_{41,app}+c_{51,app}}\approx \frac{k_{4}c_{41}\left(1+[A]/K_{dA,4}\right)}{c_{41}\left(1+[A]/K_{dA,4}\right)}=k_{4}$$

remains roughly unchanged and is not listed in Fig 2 above since the primary goal of mechanism-based activation is to improve catalytic efficiency, which does not depends on *v*_*max*_.

### Conditions for mechanism-based activation

We now consider thermodynamic conditions on the binding of a modulator A for mechanism-based sirtuin activation under the rapid equilibrium segments approximation, along with the expected changes in the steady state, equilibrium and dissociation constants in the sirtuin reaction mechanism.

First, as noted above, *v*_*max* /_ [*E*]_0_ is roughly unchanged within this family of mechanisms as long as the *K*_*d*,*A*_’s for [A] binding to the various represented complexes in the reaction mechanism satisfy condition (iii). This is reasonable as long as the modulator does not lead to a significant increase in coproduct binding affinity and reduction in coproduct dissociation rate (for example, the stabilization of a closed loop conformation). As in the case of Ex-527 [23], the latter can render product dissociation rate limiting and reduce k_cat_. If k_cat_ is reduced by the modulator, the net extent of activation will be reduced. However, even if this is the case, reduction in k_cat_ will not affect catalytic efficiency k_cat_/*K*_*m*_. Hence it is justified to omit binding of A to the coproduct complex from the mechanism-based activation model. Moreover, binding of A will not substantially reduce k_cat_ if the rate limiting chemistry step is much slower than product release.

The goal of mechanism-based activation is to increase k_cat,app_/*K*_*m*,*app*_. According to Fig 2b, $K_{m,NAD^{+},app}$ will be smaller than $K_{m,NAD^{+}}$ if *K*_*d*1,*A*_ / *K*_*d*4,*A*_ ≥ (*K*_*d*1,*A*_ / *K*_*d*2,*A*_)(*K*_*d*2,*A*_ / *K*_*d*3,*A*_)(*K*_*d*3,*A*_ / *K*_*d*4,*A*_) > 1.

To identify mechanisms by which this can occur in terms of the steps in the sirtuin-catalyzed reaction, we consider in turn each of these three respective ratios of *K*_*d*,*A*_’s (or equivalently, the ΔΔ*G*’s of the NAD^+^ binding, exchange, and NAM binding reactions as implied by Eq (7)) induced by A binding.

According to Fig 2e, *K*_d1,A_ / *K*_d2,A_ < 1 would imply that A binding increases the binding affinity of NAD^+^ to the E.Ac-Pr complex. This is the primary means by which allosteric activators enhance activity, but not the only possibility for mechanism-based activation. In principle, it is possible for a mechanism-based activator to reduce *K*_*m*,*NAD+*_ even if *K*_d1,A_ ≥ *K*_d2,A_. In this case, in order to have $K_{m,NAD^{+},app}<K_{m,NAD^{+}}$, we require (*K*_*d*2,*A*_/*K*_*d*3,*A*_)(*K*_*d*3,*A*_/*K*_*d*4,*A*_) > *K*_*d*1,*A*_/*K*_*d*2,*A*_ or equivalently, according to (8), (*K*_*d*,*NAM*_′/*K*_*ex*_′)(*K*_*ex*_/*K*_*d*,*NAM*_) > *K*_*d*,*NAD+*_′/*K*_*d*,*NAD+*_. The decrease in $K_{m,NAD^{+}}$ can then be due to modulation of the exchange rate constants that induces a decrease in *K*_*ex*_, an increase in *K*_*d*,*NAM*_, or both. If *K*_*d*,*NAM*_ changes in the presence of modulator, this corresponds to mixed noncompetitive inhibition [35] of base exchange (Fig 1).

As we have previously shown [35], the NAM moiety of NAD^+^ engages in nearly identical interactions with the enzyme before and after bond cleavage. The salient difference is a conformational change in a conserved phenylalanine side chain (e.g., Phe33 in Sir2Tm, Phe157 in SIRT3) that destabilizes NAM binding after bond cleavage [40, 41]. Since NAM binding is already destabilized by the native protein conformation in this way, and since under rapid NAM dissociation (approximation iii above, which is believed to hold for sirtuins [38]) k_2_ and k_-2_ do not appear in the expression for *K*_*m*_, $\frac{K_{d2,A}}{K_{d3,A}}$ is likely to make the dominant contribution to $\frac{K_{d2,A}}{K_{d4,A}}$ for activators. Although the value of *K*_*d*2_ / *K*_*d*4_ required for activation is likely to be achieved primarily by altering the free energy change of the NAM cleavage reaction, our model accommodates the possibility of arbitrary combinations of ΔΔ*G*_*ex*_ and ΔΔ*G*_*bind*,*NAM*_.

In our original model for sirtuin kinetics in S2 Fig, we assumed that both *K*_*d*,*NAM*_’s—namely, those for dissociation of NAM from E.Ac-Pr.NAM and E.ADPR-Pr-Im.NAM—are roughly equal. We maintain this condition in the presence of A binding, which is reasonable given that A is assumed to not interact directly with the peptide or ADPR moiety. Hence, we have:

$$\frac{\left[E.ADPR-\text{Pr}-\text{Im}][\text{NAM}\right]}{\left[E.ADPR-\text{Pr}-\text{Im}.NAM\right]}\approx \frac{\left[E.Ac-\text{Pr}][\text{NAM}\right]}{\left[E.Ac-\text{Pr}.NAM\right]}\Leftrightarrow K_{d5,A}\approx \frac{K_{d1,A}K_{d3,A}}{K_{d4,A}}$$ (9)

Returning to the expression in Fig 2a for $K_{m,NAD^{+},app}$ and substituting (1+[*A*]/*K*_*d*2,*A*_)/(1+[*A*]/*K*_*d*1,*A*_) ≥ 1, the rapid equilibrium assumptions applied to the present system imply that in order to activate the enzyme at [NAM] = 0, if A does not improve cofactor binding affinity it must increase k_1_ (k_1,app_>k_1_), k_2_ (k_2,app_>k_2_) or both. The rapid equilibrium segments model is not able to distinguish between these scenarios. If A increases $K_{d,NAD^{+},app}$, it is unlikely that an increase k_1_ will achieve activation. An increase in k_2_ implies acceleration of the rate of NAM cleavage. In the rapid equilibrium segments framework, this occurs through preferential stabilization of the E.ADPR-Pr-Im complex. Note that across all sirtuins studied, NAM cleavage induces structural changes (for example, unwinding of a helical segment in the flexible cofactor binding loop [42, 43]) and such changes might enable preferential stabilization of the E.ADPR-Pr-Im complex, in a manner similar to the stabilization of specific loop conformations by mechanism-based inhibitors [23]. Indeed, stabilization of alternative, non-native conformations of this loop have been observed crystallographically by reported activators of sirtuins other than SIRT3, including both long-chain fatty acids [38, 44] and recently reported activators of SIRT5 and SIRT6 [30]. We discuss below the biophysical underpinnings whereby an increase in a forward rate constant could be achieved through preferential stabilization of the intermediate complex.

Given that an open loop conformation favors NAD^+^ binding, if a closed loop conformation is stabilized by the modulator, we expect the following thermodynamic conditions on the binding of A to the various complexes in the sirtuin reaction mechanism:

$${\text{K}}_{d1,A}\leq K_{d2,A}↔K_{d,NAD+}^{\prime }\geq K_{d,NAD+}$$ (10)

$$K_{d2,A}\gg K_{d3,A}↔K_{ex}^{'}\ll K_{ex}$$

where the >> sign signifies that *K*_*d*2,*A*_/*K*_*d*3,*A*_ > *K*_*d*1,*A*_ / *K*_*d*2,*A*_. We also require the necessary but not sufficient condition that *K*_*d*2,*A*_/ *K*_*d*4,*A*_ > *K*_*d*1,*A*_ / *K*_*d*2,*A*_. As noted above, increasing *K*_*d*3,*A*_/*K*_*d*4,*A*_ (which would destabilize NAM binding) is not considered as a mode of activation since NAM is believed to already dissociate quickly from the native active sites of sirtuins. Within conventional nomenclature, decrease in *K*_*ex*_ corresponds to hyperbolic (or partial) noncompetitive inhibition [35] of base exchange/activation of NAM cleavage (as opposed to complete quenching of the base exchange reaction; see Fig 1).

We now consider the effects of binding of such a modulator A that favors a closed cofactor loop conformation on the remaining steady state constants.

- *K*_1,*app*_: Reduction in *K*_1_, which is associated with a reduction in *K*_*d*3,*A*_/*K*_*d*4,*A*_ (Fig 2d), can increase *K*_*m*,*NAD+*_. A modulator has more favorable properties if *K*_1_ does not decrease significantly, but as discussed above, an increase in *K*_1_ will generally not be sufficiently for activation. Moreover, if *K*_3_ increases in the presence of modulator, a decrease in *K*_1_ may not be consequential.
- *K*_2,*app*_: With conditions (10), the expression in Fig 2c predicts a limited change in *K*_2_.
- *K*_3,*app*_: According to Fig 2b, in the presence of such a mechanism-based activator, *K*_3_ is expected to increase by a factor more than that for *K*_2_ under the rapid equilibrium segments approximation. This can occur due to an increase in *K*_*d*,*NAM*_, a decrease in *K*_*ex*_, or both.
- *α*_*app*_: According to the equations in Fig 2b and 2c, the aforementioned condition that *K*_*d*2,*A*_/*K*_*d*4,*A*_ ≥ *K*_*d*1,*A*_ / *K*_*d*2,*A*_ implies an increase in *α*.

**Fig 3:**
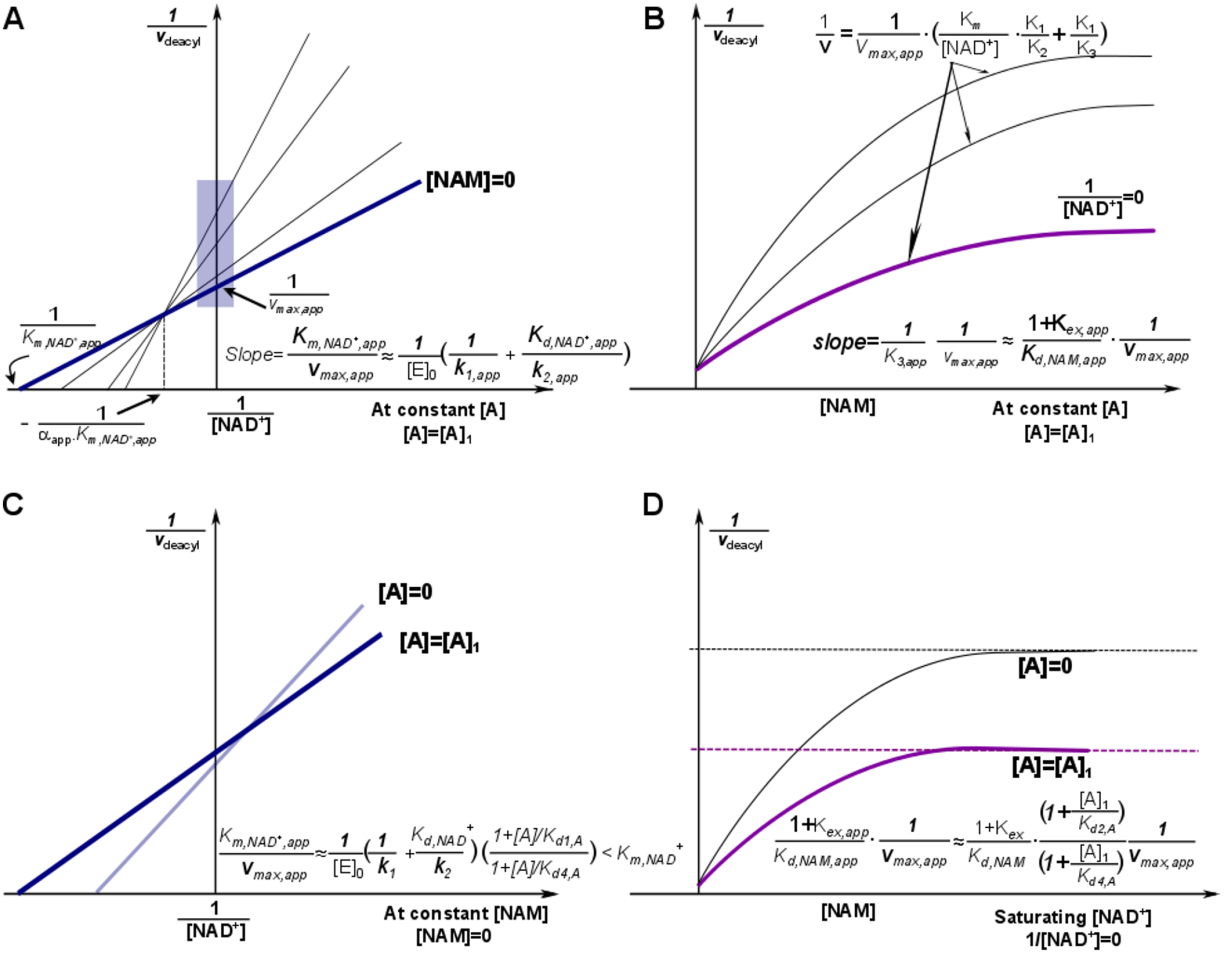
Mechanism-based activation of sirtuin enzymes: predicted steady-state properties and dose-response behavior. **(A)** Double reciprocal plots for deacylation initial rate measurements in the presence of activator. The blue box on the y-axis highlights the data that is used to construct the Dixon plot at saturating [NAD^+^] depicted in (B). **(B)** Dixon plots for deacylation initial rate measurements in the presence of activator. The arrows point to predicted plateaus in these curves. **(C)** Comparison of double reciprocal plots at [NAM] = 0 μM in the presence and absence of activator. **(D)** Comparison of Dixon plots at 1/[NAD^+^] = 0 in the presence and absence of activator. “A” denotes a mechanism-based sirtuin activating compound. Note that the model depicted omits the term quadratic in [NAM] in Eq (1) and the plateaus/dotted lines shown in the Dixon plots are the asymptotic values to which the model-predicted rates converge in the absence of this term.

Fig 3 depicts the model-predicted changes to the various steady state, Michaelis and dissociation constants in the sirtuin reaction mechanism in the presence of such a modulator.

The conditions for activation described above do not need to hold for a hit compound for mechanism-based activation. A hit compound may be defined as one that satisfies a subset of the conditions enumerated above, and may also display comparatively little inhibition in the pre-steady state burst phase. Such compounds may be capable of undergoing further improvement for substrate-specific activation of sirtuins like SIRT3, under physiologically relevant NAD^+^ depletion conditions. For example, it is possible that catalytic efficiency does not increase in the absence of NAM, but does so in its presence (for example, due to increase in the *K*_*3*_ parameter above). Note that due to nonzero physiological concentration of NAM in the cell, reduction of NAM inhibition can also contribute to activation under physiologically relevant conditions. Alternatively, the relative rates of deacylation in the presence and absence of modulator could converge under certain combinations of [NAD^+^] and [NAM].

### Potential means of increasing k_2_

From the standpoint of chemical mechanisms of activation, the theory presented raises the important question of how the NAM cleavage rate k_2_ of sirtuins can be accelerated by a ligand that binds to the various complexes in the deacylation reaction with the specified relative affinities, as predicted by equation in Fig 2a. It is important to note in this regard that the NAM cleavage reaction in sirtuins is generally believed to be endothermic, which enables effective NAM inhibition of the reaction [32, 45]. Unlike exothermic reactions, stabilization of products in endothermic reactions can decrease the activation barrier for the forward reaction, due to the fact that the transition state resembles the products more than the reactants. The energetics of this reaction, including the role of protein conformational changes, are being studied computationally in our group for mammalian sirtuins.

## Discussion

We have presented a model for activation of sirtuin enzymes suitable for the design and characterization of MB-STACs. Using this modeling framework, we have shown how modulation of $K_{m,NAD^{+}}$ can in principle increase the activity of sirtuins. This activation can also apply in the presence of NAM, decreasing the sensitivity of the sirtuin to physiological NAM inhibition in addition to increasing its sensitivity to physiological NAD^+^. The mechanism of action of recently reported nonallosteric activators of sirtuins, such as honokiol (HKL) [28], which was reported to be a SIRT3 activator, and other compounds recently reported to activate SIRT5 and SIRT6 [30], can be characterized using the methods presented.

The rapid equilibrium segments approximation (Fig 2) was applied in order to illustrate how a ligand that binds outside the NAD^+^ binding site can in principle increase sirtuin activity through only modulation of the relative free energies of the various species in the reaction mechanism. More detailed analysis of the mechanism of action of hit compounds for mechanism-based enzyme activation can be achieved by complete kinetic characterization in presence/absence of the activator, as discussed further below.

Structurally, binding outside of the NAD^+^ binding site (the so-called A and C pockets [31, 46]) appears to be essential for mechanism-based activation. For example, consider the binding sites of long-chain fatty acids and Ex-527 [23, 44]. Rational design will require analysis of the relative free energies of complexes depicted in Fig 1. We have recently initiated computational studies [35] that assess such free energy differences for some of the front face (apo) complexes in this Fig 1, and further studies are in progress.

Structure-activity relationships for mechanism-based enzyme activating compounds are very different from those for allosteric activators. The kinetic effects of allosteric activators that operate through either reduction of substrate *K*_*d*_ or increase in k_cat_ are mediated through global conformational changes that generally do not involve competing effects on steady state constants. Molecules that bind to allosteric sites will generally bind differentially to the intermediates in a predetermined way (with specified *K*_*d*_ ratios) irrespective of their structural details; the same conformational change is induced by binding, irrespective of structure.

In mechanism-based activation, the kinetic effects of the activator depend on modulation of local degrees of freedom in the active site, not global conformational changes described by principal components. Typically, mechanism-based activators may alter the distributions of local degrees of freedom whose conformations change during the course of the reaction, which leads to tradeoffs in the ΔΔG's for various reactions steps upon stabilization of one such conformation. As discussed above, activators of sirtuin enzymes that do not possess an allosteric site [13, 30, 44] have been shown crystallographically to induce changes in the conformation of the flexible cofactor binding loop in sirtuin enzymes. This loop changes conformation after the first chemical step of the reaction, namely the cleavage of nicotinamide from the NAD^+^ cofactor. In these cases, the local degrees of freedom above are the backbone and side chain degrees of freedom in the flexible cofactor binding loop. Perhaps the most detailed structural studies on conformational changes in sirtuins induced by modulators have been reported for the mechanism-based inhibitor Ex-527, discussed above.

By inducing changes in the probability distributions of local active site degrees of freedom (DOF), the differential binding affinities of an activator (“A”) to the various intermediates in the catalytic mechanism induce effects on the free energy differences ΔΔG's) in that mechanism. We have already considered above the effect of arbitrary concentrations of a modulator A on activity given specified *K*_*di*,*A*_*'s* of modulator binding to different intermediates. The impact of a modulator A on the free energy differences relevant to catalytic mechanism can be represented in terms of a potential of mean force (PMF) that alters the probabilities of various receptor states in the conformational ensemble, and hence alters the catalytic free energy differences. The PMF only changes due to deviations of the specified local DOF, and can be calculated for any given modulator structure by using the corresponding molecular Hamiltonian to evaluate the average interaction potential in terms of these local DOF. Then, the effect of the modulator A on the binding free energy of a ligand (e.g., substrate) can be expressed in terms of the exponential mean of the PMF, providing structure-activity relations solely in terms of this PMF. Details of these calculations will be provided in future work.

## Conclusions

The enzyme activation theory presented herein enables the identification and evolution of important hits that may be inhibitors, not activators, by decomposing the observed kinetic effects of a modulator into components and identifying those molecules that display favorable values of a subset of these components as hits even if the net effect on catalytic turnover is inhibition. Such workflows would be fundamentally different from traditional drug discovery workflows and would bear more similarity to the directed evolution of enzymes. The theory presented also establishes foundations for the rational design of sirtuin-activating compounds, enabling the application of state-of-the-art computational methods to activator design in a manner analogous to computational enzyme design [47]. Once crystal structures solved, enzyme engineering methods can be used for hit to lead evolution based on the above theory.

To evolve such hit compounds into enzyme activators, computational design methods using high-resolution protein force fields [35, 47] can be applied in conjunction with experimental learning algorithms, such as directed evolution. Typically, the overall catalytic activity is used in directed evolution algorithms because it is the only quantity that can be efficiently measured in the laboratory. The ability to rapidly estimate the multiple free energy differences that determine catalytic activity through system identification methods [36] would allow multiobjective directed evolution algorithms to be applied in the laboratory. With system identification, the parameter estimates can be used with such evolutionary algorithms to sample the nondominated Pareto frontier of Hamiltonian designs [48].

A framework for catalyst system identification was proposed in [36]. This framework can be applied in the context of hit-to-lead evolution for mechanism-based activators, where the Hamiltonian is iteratively updated and catalytic free energy differences automatically estimated for each modulator Hamiltonian. The catalyst system identification framework can be automated using programmable logic and fluidic control systems in order to achieve a high-throughput implementation, as described in our recent publication [36].

In computational modulator design [47], where focused libraries of mutations may be generated, free energy differences calculated from mixed quantum/molecular mechanics (QM/MM) methods for reactive chemistry and free energies of binding for substrate/product binding calculated using free energy perturbation can be compared to free energy data obtained using system identification for all steps of the catalytic mechanism. Computationally efficient predictions of free energy differences, especially those for binding events, must be calibrated through correlations with experimental data. The experimental estimates rapidly provided by system identification for any catalytically relevant free energy difference can be used for calibration.

Once the catalyst without Hamiltonian modulation (e.g., the wild-type enzyme) is accurately characterized, all other modulated systems can be characterized in terms of the ratios of k_i'_s or equivalently the ΔΔGs, which are used in high-throughput evolutionary algorithms as described in [36].

## Appendix A1

### Steady state parameters of sirtuin-catalyzed deacylation expressed in terms of rate constants in the enzymatic reaction mechanism (Eq (2))

*Kex* = k_-2_/k_2_, and the approximations refer to the case where *k*_*4*_*≪k*_*j*_, *j≠* 4.

$$v_{max}=\frac{k_{4}*k_{2}k_{-3}(k_{-3}+k_{4})}{k_{-3}k_{2}k_{-3}+k_{4}(k_{-3}k_{4}+k_{-3}k_{-3}+k_{-3}k_{-2}+k_{-3}k_{2}+k_{2}k_{4})}{\left[E\right]}_{0}\approx k_{4}{\left[E\right]}_{0}$$ (11)

$$K_{m,NAD^{+}}=\frac{v_{max}}{{\left[E\right]}_{0}}\frac{k_{2}k_{-3}+k_{-1}k_{-3}+k_{-1}k_{-2}+k_{2}k_{4}+k_{-1}k_{4}}{k_{1}k_{2}(k_{-3}+k_{4})}\approx k_{4}\left(\frac{1}{k_{1}}+K_{d,NAD^{+}}\frac{k_{-3}+k_{-2}}{k_{-3}k_{2}}\right)$$ (12)

$$\frac{1}{K_{1}}=\frac{k_{3}}{k_{-3}+k_{4}}\approx \frac{1}{K_{d,NAM}}$$ (13)

$$\frac{1}{K_{2}}=\frac{1}{K_{m,NAD^{+}}}\frac{k_{3}[k_{-1}(k_{-2}k_{-3}+k_{4}k_{-2}+2k_{4}k_{-3})+k_{4}(k_{2}k_{4}+k_{-1}k_{4}+2k_{-3}k_{2})]}{k_{1}[k_{-3}k_{2}k_{-3}+k_{4}(k_{-3}k_{4}+k_{-3}k_{-3}+k_{-3}k_{-2}+k_{-3}k_{2}+k_{1}k_{4})]}\approx \frac{K_{d,NAD^{+}}K_{ex}}{K_{m,NAD^{+}}K_{d,NAM}}$$ (14)

$$\frac{1}{K_{1}}\equiv \frac{1}{\alpha K_{2}}=\frac{k_{3}\left[k_{-3}\left(k_{-2}+k_{2}+k_{4}\right)+k_{4}k_{2}\right]}{k_{-3}\left[k_{-3}k_{2}+k_{4}k_{4}+k_{-3}k_{4}+k_{4}k_{-2}+k_{4}k_{2}\right]+k_{4}k_{4}k_{2}}\approx \frac{1+K_{ex}}{K_{d,NAM}}$$ (15)

## Appendix A2

Expressions for c_ij_’s in Eq (1):

$$c_{11}=k_{4}k_{-3}\left[k_{4}k_{2}+k_{4}k_{-1}+k_{2}k_{-3}+k_{-1}k_{-3}+k_{-2}k_{-1}\right]$$ (16)

$$c_{12}=k_{3}k_{-2}k_{-1}k_{-2}^{}+k_{4}\left(k_{2}k_{-3}k_{3}+k_{-1}^{}k_{-3}k_{3}\right)$$ (17)

$$c_{21}=k_{4}\left(k_{-3}k_{1}k_{4}+k_{-3}k_{1}k_{-3}+k_{-3}k_{1}k_{-2}\right)$$ (18)

$$c_{22}=k_{1}k_{3}k_{-2}k_{-3}+k_{4}k_{1}k_{3}k_{-3}$$ (19)

$$c_{31}=k_{4}k_{1}k_{2}k_{-3}$$ (20)

$$c_{32}=k_{1}k_{2}k_{3}k_{-3}$$ (21)

$$c_{41}=k_{1}k_{2}k_{-3}k_{-3}$$ (22)

$$c_{51}=k_{4}k_{1}k_{2}k_{4}$$ (23)

$$c_{52}=k_{4}\left(k_{4}k_{3}k_{2}+k_{4}k_{-1}k_{3}+k_{-3}k_{3}k_{2}+k_{-2}k_{-1}k_{3}+k_{-2}k_{-1}k_{3}\right)$$ (24)

$$c_{53}=k_{4}k_{1}k_{2}k_{3}$$ (25)

$$c_{54}=k_{-2}k_{-1}k_{3}k_{3}+k_{4}\left(k_{2}k_{3}k_{3}+k_{-1}k_{3}k_{3}\right)$$ (26)

## Supporting information

S1 Fig Chemical mechanism of sirtuin-catalyzed deacylation and modes of sirtuin activation. Following sequential binding of acylated peptide substrate and NAD^+^ cofactor, the reaction proceeds in two consecutive stages: i) cleavage of the NAM moiety of NAD^+^ (ADP-ribosyl transfer) through the nucleophilic attack of the acetyl-Lys side chain of the protein substrate to form a positively charged O-alkylimidate intermediate, and ii) subsequent formation of deacylated peptide. For simplicity, all steps of stage ii as well as AADPR + Pr dissociation are depicted to occur together with rate limiting constant *k*_*4*_. The schematic highlights mechanism-based activation through NAD^+^ *K*_*m*_ reduction rather than the *K*_*d*_ peptide reduction that known allosteric sirtuin activators elicit.
(TIF)

S2 Fig General model for sirtuin-catalyzed deacylation in the presence of NAD^+^ and NAM. This provides a minimal kinetic model that captures the essential features of sirtuin deacylation kinetics suitable for predicting the effects of mechanism-based modulators on sirtuin activity. In the presence of saturating Ac-Pr, E is rapidly converted into E.Ac-Pr and NAM binding to E can be neglected, resulting in a simplified reaction network with 5 species. Ac-Pr, acetylated peptide; ADPR, adenosine diphosphate ribose; AADPR, O-acetyl adenosine diphosphate ribose.
(TIF)

S1 File Steady state constant and rapid equilibrium segments expressions. (DOCX)
