# Supplementary figures and images for: Biophysical characterization of hit compounds for mechanism-based enzyme activation
Source: PLoS One. 2018 Mar 16;13(3):e0194175. doi: 10.1371/journal.pone.0194175 (PMC5856274; doi:10.1371/journal.pone.0194175)

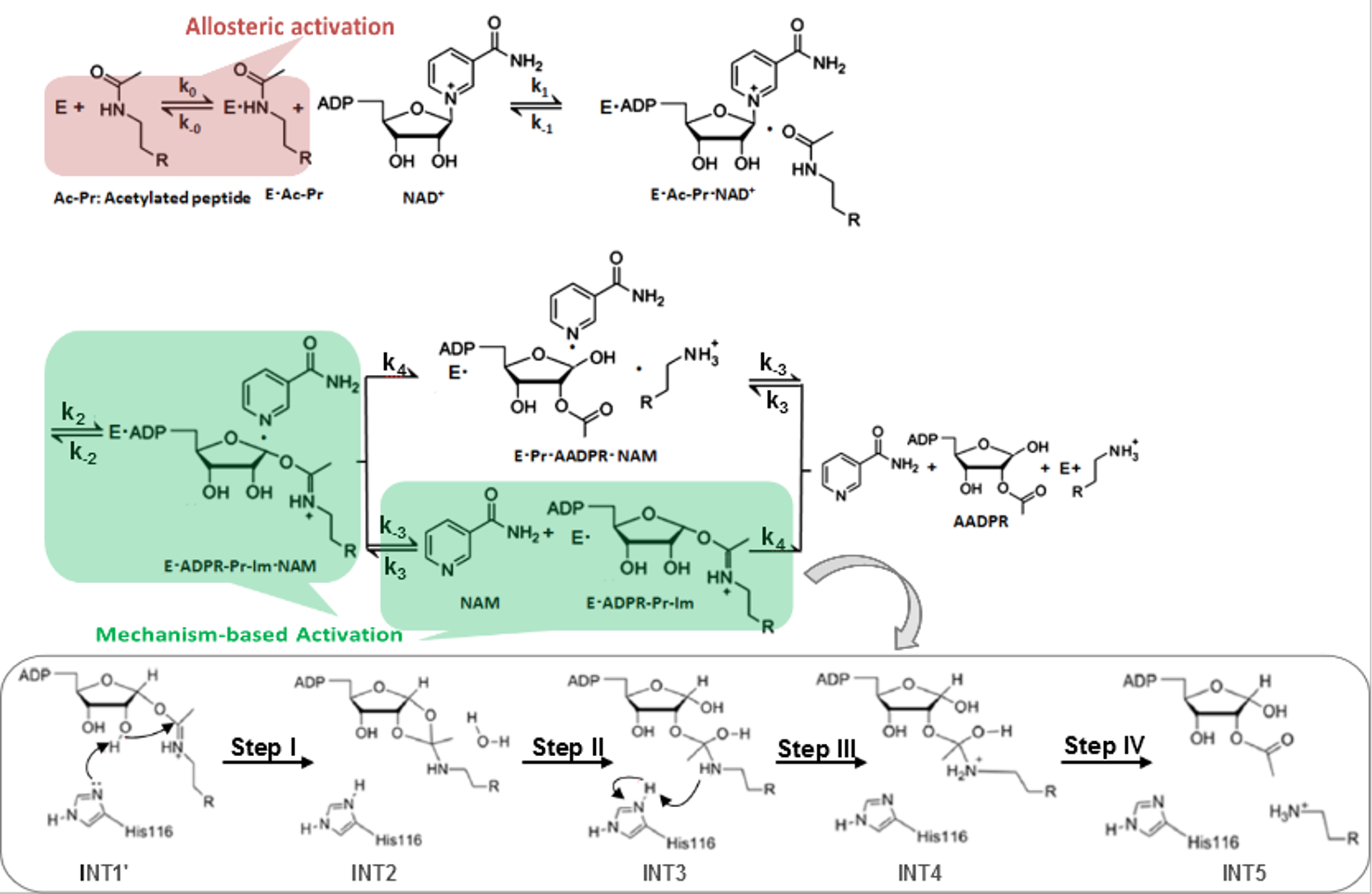

Supplement: S1 Fig — Following sequential binding of acylated peptide substrate and NAD+ cofactor, the reaction proceeds in two consecutive stages: i) cleavage of the NAM moiety of NAD+ (ADP-ribosyl transfer) through the nucleophilic attack of the acetyl-Lys side chain of the protein substrate to form a positively charged O-alkylimidate intermediate, and ii) subsequent formation of deacylated peptide. For simplicity, all steps of stage ii as well as AADPR + Pr dissociation are depicted to occur together with rate limiting constant k4. The schematic highlights mechanism-based activation through NAD+ Km reduction rather than the Kd peptide reduction that known allosteric sirtuin activators elicit. (TIF) [file pone.0194175.s001.tif]

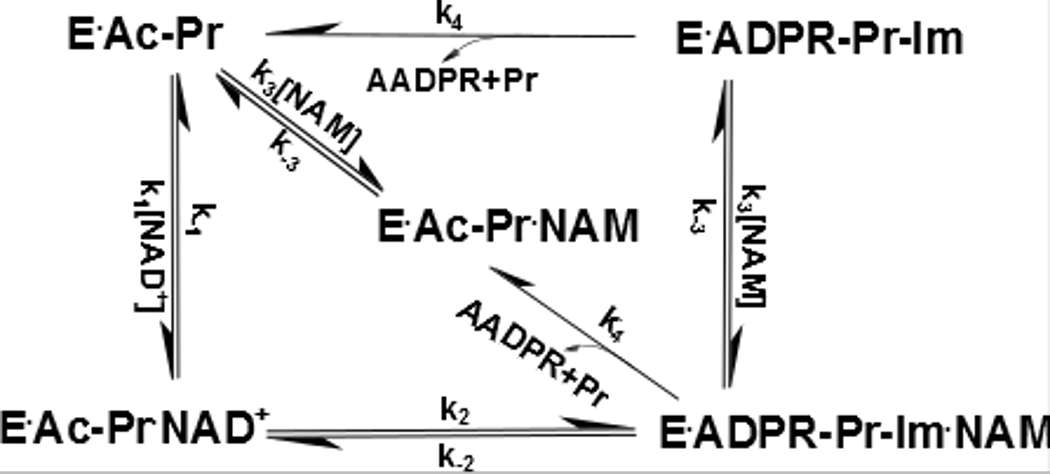

Supplement: S2 Fig — This provides a minimal kinetic model that captures the essential features of sirtuin deacylation kinetics suitable for predicting the effects of mechanism-based modulators on sirtuin activity. In the presence of saturating Ac-Pr, E is rapidly converted into E.Ac-Pr and NAM binding to E can be neglected, resulting in a simplified reaction network with 5 species. Ac-Pr, acetylated peptide; ADPR, adenosine diphosphate ribose; AADPR, O-acetyl adenosine diphosphate ribose. (TIF) [file pone.0194175.s002.tif]
