# Supplementary material for: Biophysical characterization of hit compounds for mechanism-based enzyme activation
Source: PLoS One. 2018 Mar 16;13(3):e0194175. doi: 10.1371/journal.pone.0194175 (PMC5856274; doi:10.1371/journal.pone.0194175)
Supplement: S1 File — (DOCX) [file pone.0194175.s003.docx]

**Supporting Information**

**Steady state constant and rapid equilibrium segments expressions**

The expressions for the steady state constants in Appendix A1 (11-15) follow from

$$v_{\max}= \frac{k_{4}\left(c_{31}+c_{41}\right)}{c_{21}+c_{31}+c_{41}+c_{51}}[E]_{0}$$

$$K_{m,NAD^{+}}=\frac{c_{11}}{c_{21}+c_{31}+c_{41}+c_{51}}$$

$\frac{1}{K_{1}}=\frac{c_{32}}{c_{31}+c_{41}}$ (A3)

$$\frac{1}{K_{2}}=\frac{1}{K_{m,\mathrm{NAD}^{+}}}\frac{c_{12}+c_{52}}{c_{21}+c_{31}+c_{41}+c_{51}}$$

$$\frac{1}{K_{2'}}=\frac{1}{K_{m,\mathrm{NAD}^{+}}}\frac{c_{54}}{c_{21}+c_{31}+c_{41}+c_{51}}$$

$$\frac{1}{K_{3}}=\frac{c_{22}+c_{32}+c_{53}}{c_{21}+c_{31}+c_{41}+c_{51}}$$

The rapid equilibrium segments expressions for the steady-state concentrations of the species in the sirtuin reaction mechanism depicted in S1 Fig are:

$$[E.Ac-\Pr]/[E.Ac-\Pr]_{\begin{matrix} 0 \\ \end{matrix}}\approx c_{11}+c_{12}[\mathrm{NAM}]$$

$$[E.Ac-\Pr.\mathrm{A}]/[E.Ac-\Pr]_{\begin{matrix} 0 \\ \end{matrix}}\approx\frac{[A]}{K_{d1,A}}\left(c_{11}+c_{12}[\mathrm{NAM}]\right)$$

$$[E.Ac-\Pr.\mathrm{NAD}^{+}]/[E.Ac-\Pr]_{\begin{matrix} 0 \\ \end{matrix}}\approx c_{21}[NAD^{+}]+c_{22}[\mathrm{NAD}^{+}][\mathrm{NAM}]$$

$[E.Ac-\Pr.\mathrm{NAD}^{+}.\mathrm{A}]/[E.Ac-\Pr]_{\begin{matrix} 0 \\ \end{matrix}}\approx\frac{[A]}{K_{d2,A}}\left(c_{21}[NAD^{+}]+c_{22}[\mathrm{NAD}^{+}][\mathrm{NAM}]\right)$ (A4)

$$[E.ADPR-Ac-\mathrm{Im}.NAM]/[E.Ac-\Pr]_{\begin{matrix} 0 \\ \end{matrix}}\approx c_{31}[NAD^{+}]+c_{32}[NAD^{+}][NAM]$$

$$[E.ADPR-Ac-\mathrm{Im}.NAM.\mathrm{A}]/[E.Ac-\Pr]_{\begin{matrix} 0 \\ \end{matrix}}\approx\frac{[A]}{K_{d3,A}}\left(c_{31}[NAD^{+}]+c_{32}[NAD^{+}][NAM]\right)$$

$$[E.ADPR-Ac-\mathrm{Im}]/[E.Ac-\Pr]_{\begin{matrix} 0 \\ \end{matrix}}\approx c_{41}[\mathrm{NAD}^{+}]$$

$$[E.ADPR-Ac-\mathrm{Im}.\mathrm{A}]/[E.Ac-\Pr]_{\begin{matrix} 0 \\ \end{matrix}}\approx\frac{[A]}{K_{d4,A}}\left(c_{41}[\mathrm{NAD}^{+}]\right)$$

$$[E.\mathrm{Ac}-\Pr.NAM]/[E.Ac-\Pr]_{\begin{matrix} 0 \\ \end{matrix}}\approx c_{51}[NAD^{+}]+c_{52}[NAM]+\mathrm{c}_{53}[\mathrm{NAD}^{+}][\mathrm{NAM}]$$

$$[E.\mathrm{Ac}-\Pr.NAM.\mathrm{A}]/[E.Ac-\Pr]_{\begin{matrix} 0 \\ \end{matrix}}\approx\frac{[A]}{K_{d5,A}}\left(c_{51}[NAD^{+}]+c_{52}[NAM]+\mathrm{c}_{53}[\mathrm{NAD}^{+}][\mathrm{NAM}]\right)$$
